# Correlates of Sleep Health among Older-Age People with and without HIV in Uganda

**DOI:** 10.1007/s10461-024-04512-x

**Published:** 2024-10-08

**Authors:** Moka Yoo-Jeong, Aneeka Ratnayake, Yao Tong, Alexander C. Tsai, Robert Paul, Zahra Reynolds, Christine S. Ritchie, Janet Seeley, Susanne S. Hoeppner, Flavia Atwiine, Samson Okello, Noeline Nakasujja, Deanna Saylor, Meredith Greene, Stephen Asiimwe, Edna Tindimwebwa, Jeremy Tanner, Brianne Olivieri-Mui, Mark J. Siedner

**Affiliations:** 1 School of Nursing, Bouvé College of Health Sciences, Northeastern University, Boston, USA MA; 2 The Roux Institute, Northeastern University, Portland, USA ME; 3 Medical Practice Evaluation Center, Mongan Institute, Massachusetts General Hospital, Boston, USA MA; 4 Harvard Medical School, Boston, USA MA; 5 Center for Global Health, Massachusetts General Hospital, Boston, USA MA; 6 Mbarara University of Science and Technology, Mbarara, Uganda; 7 Department of Psychological Sciences, University of Missouri - St Louis, St Louis, USA MO; 8 Division of Palliative Care and Geriatric Medicine, Department of Medicine, Massachusetts General Hospital, Boston, USA MA; 9 Center for Aging and Serious Illness, Mongan Institute, Massachusetts General Hospital, Boston, USA MA; 10 Department of Global Health and Development, Faculty of Public Health and Policy, London School of Hygiene & Tropical Medicine, London, UK; 11 School of Public Health, Harvard T.H.Chan, Boston, USA MA; 12 Department of Psychiatry, College of Health Sciences, Makerere University, Kampala, Uganda; 13 Department of Neurology, Johns Hopkins University School of Medicine, Baltimore, USA MD; 14 Department of Medicine, Indiana University School of Medicine, Bloomington, USA IN; 15 Indiana University Center for Aging Research at the Regenstrief Institute, Indianapolis, USA IN; 16 Kabwohe Clinical Research Centre, Kabwohe, Uganda; 17 Biggs Institute for Alzheimer’s and Neurodegenerative Diseases, University of Texas Health Science Center, San Antonio, USA TX; 18 Department of Health Sciences, Bouvé College of Health Sciences, Northeastern University, Boston, USA MA; 19 The Marcus Institute for Aging Research, Hebrew SeniorLife, Harvard Medical School, Boston, USA MA; 20 Department of Epidemiology, University of North Carolina, Chapel Hill, NC USA

**Keywords:** Sleep, Older Adults, Uganda, HIV, Depression

## Abstract

There is a growing population of older people with HIV (PWH) in Uganda. Sleep problems disproportionately affect older people and PWH. This study aimed to estimate correlates of sleep health among older Ugandans (aged ≥ 50 years) with and without HIV, using data from the Quality of Life and Aging with HIV in Rural Uganda Study. We used the Pittsburgh Sleep Quality Index to assess sleep quality, duration, and efficiency. We fitted multivariable linear and logistic regression models to estimate the associations between sleep outcomes and variables selected based on the Senescent Sleep Model: age, HIV serostatus, loneliness, urbanicity, symptoms of depression and anxiety, and perceived stress. Of 556 participants, 271 were PWH and 285 were people without HIV (PWoH). There were no statistically significant differences in sleep outcomes by HIV serostatus. Of the total sample, most reported very good (32.79%) or fairly good sleep quality (49.37%). The mean sleep duration was 6.46 h (SD = 1.74). The mean sleep efficiency was 73.98% (SD = 19.52%) with 36.69% having optimal (≥ 85%) sleep efficiency. A positive depression screen was associated with worse sleep quality (adjusted odds ratio [aOR] = 0.21; 95% CI [0.12, 0.36]), shorter sleep duration (b=-0.44; 95% CI [-0.60, -0.28]), and worse sleep efficiency (aOR = 0.51; 95% CI[0.31, 0.83]). Interventions targeting depression may improve sleep among older Ugandans, independent of HIV serostatus. Longitudinal studies are needed to determine the potential bidirectionality of this relationship and elucidate pathways to support sleep health among older Ugandans.

## Introduction

In Uganda, people aged 50 years and older comprised 7% of the population in 2020 [1], and it is projected that this proportion will grow rapidly. This trend parallels the shift in demographic characteristics of people with HIV (PWH), where the number of older PWH in Uganda is also increasing [2] due to increased access to HIV care, including early diagnosis and initiation of suppressive antiretroviral therapy (ART). With the aging of the population of PWH and people without HIV (PWoH), sleep health is expected to be an increasingly important public health concern, because it disproportionately affects both older people and PWH [3–5].

Poor sleep health is an underappreciated public health concern that is strongly associated with mortality and morbidity [6, 7]. Poor sleep health is common with advancing age, in part, due to other age-related comorbidities, symptoms, and life course transitions that affect overall health [8]. In the SAGE Well-Being of Older People Study of community-dwelling older people in South Africa and Uganda, sleep difficulties were widely reported in both settings and were correlated with depressive symptoms in the Ugandan sample [9]. This study did not explore other dimensions of sleep health such as sleep duration or efficiency, nor did the investigation on the effect of HIV serostatus. The unexplored dimension of HIV is important given that PWH are disproportionately affected by sleep problems [3, 4] and by depression [10].

Sleep health can be characterized by multiple dimensions, including sleep quality, duration, and efficiency [11]. *Sleep quality* indicates an individual’s perception of satisfaction with their sleep [12]. *Sleep duration* measures the total number of hours of sleep reported within a 24-hour period. The recommended optimal sleep duration for adults is 7 to 8 h [13]. Both short (≤ 6 h) and long (≥ 9 h) sleep duration have been associated with poor health outcomes [6, 7, 14]. *Sleep efficiency* denotes the percentage of time in bed actually spent sleeping and is calculated based on the total sleep duration divided by the total time in bed. Presumably, this excludes non-sleep related activities in bed. Greater than 85% of sleep efficiency is considered optimal for adults [15]. These multiple facets of sleep health have been shown to associate with self-rated health and quality of life outcomes [11]. Together, these findings underscore the importance of investigating the different facets of sleep to yield a comprehensive picture of sleep health.

According to the Senescent Sleep Model [16], problems with sleep among older adults are conceptualized as a multifactorial geriatric syndrome wherein sleep and related poor health outcomes are determined by a combination of predisposing, precipitating, and perpetuating factors. *Predisposing factors* include age-related physiological changes to the sleep cycle. *Precipitating factors* refer to any chronic diseases that alter sleep including HIV serostatus. HIV contributes to sleep issues due to the effects of the virus and ART affecting the central nervous system and chronic immune activtation. For example, efavirenz has been associated with various adverse effects related to sleep [17]. Older PWH may be more susceptible to any adverse effects of ART regimens on sleep due to a comibination of comorbid health conditions, polypharmacy, and physiological changes related to aging that can alter drug pharmacokinetics and pharmacodynamics [18].

*Perpetuating factors* include psychosocial (e.g., loneliness, depressive symptoms, anxiety, stress) and environmental stressors (e.g., urban or rural setting) that may perpetuate poor sleep [16]. Studies from low- and middle-income countries and high-income countries suggest that combinations of psychosocial factors such as loneliness and depressive symptoms induce sleep complaints [16, 19]. Anxiety and perceived stress have shown bidirectional relationships with poor sleep health indices in studies conducted in various populations and across settings [20, 21]. Residential environment such as whether a person lives in an urban in comparison to rural setting may significantly affect multiple dimensions of sleep health. For example, urban residential living often exposes individuals to more noise and crowding, which results in poor sleep quality [22].

Guided by the Senescent Sleep Model as our conceptual framework, we conducted a cross-sectional study to estimate the prevalence of multiple dimensions of self-reported sleep health and to estimate the correlates of sleep health among older PWH and PWoH in Uganda. We hypothesized that older PWH will report greater problems with sleep than PWoH and that perpetuating psychosocial and environmental stressors would be significantly associated with sleep health regardless of HIV serostatus.

## Methods

### Study Sample and Data Collection

This study used the second wave of data collected in 2021–2022 from the *Quality of Life and Aging with HIV in Rural Uganda Study.* The study design and methods have been described in detail elsewhere [23, 24]. Briefly, the purpose of the parent study was to identify HIV-related determinants and key physical, cognitive, and social domains of health-related quality of life of older aged people with HIV in Rural Uganda [25]. Data were collected from 600 older ($\:\ge\:$50 years old) Ugandans, evenly divided by HIV serostatus, annually from October 2020. Eligible PWH (*n* = 298) were identified from a prior longitudinal cohort study [23, 26], had been on ART for at least three years, and were engaged in HIV care at study sites (ambulatory HIV clinics at Mbarara Regional Referral Hospital and Kabwohe Clinical Research Centre). PWoH were selected from the clinic catchment areas using population census data from a nearby rural population cohort [27] and village health team lists (*n* = 302). The PWoH sample had a similar distribution of age (within quartiles), sex, and site (Mbarara or Kabwohe). All study procedures were reviewed and approved by the institutional review committees at Mbarara University of Science and Technology and Mass General Brigham. Parent study also received clearance to conduct the study from the Uganda National Council for Science and Technology and the Research Secretariat in the President’s Office. All study participants provided written informed consent. After obtaining informed consent, data were collected by trained research assistants who received rigorous training in the ethical conduct of human subjects research. The first wave of the data (October 2020–2021) were collected via phone due to the COVID-19 pandemic. Thereafter, all visits were held in person.

## Measures

### Sleep Health Outcome Variables

We selected three primary outcomes of self-reported sleep health: sleep quality, sleep duration, and sleep efficiency, as measured by the Pittsburg Sleep Quality Index (PSQI) [28].

*Sleep Quality*. Participants indicated their level of sleep quality by responding to the question, “During the past month, how would you rate your sleep quality overall?” Responses were reported on a Likert-type scale, with 0 = very good, 1 = fairly good, 2 = fairly bad, 3 = very bad. For the regression models, we dichotomized responses into “good sleep quality” (very good, fairly good) and “poor sleep quality” (fairly bad, very bad).

*Sleep Duration* was measured as the number of hours of actual sleep reported by the participant. Participants responded to the following question: “During the past month, how many hours of actual sleep did you get at night?” Responses were given in hours. Our analyses included both continuous (in hours) and categorical measures of sleep duration. Sleep duration categories were based on published thresholds as very short (< 5 h), short (5–6 h), optimal (7–8 h), and long (≥ 9 h) sleep [13].

*Sleep Efficiency* was defined as the ratio of hours asleep to hours in bed, captured as a percentage. For the regression models, we dichotomized responses into “sub-optimal sleep efficiency” (< 85%) and “optimal sleep efficiency” (*≥* 85%), a threshold commonly used in insomnia research [12, 29].

### Primary Explanatory Variables of Interest

The primary explanatory variables of interest selected for the multivariable regression models, guided by the Senescent Sleep Model were: age, HIV status, urban or rural residence, loneliness, depressive symptoms, perceived stress, and anxiety symptoms. *Age* was measured on a continuous scale, in years. *HIV status* was determined at enrollment, with confirmatory HIV testing for all participants. *Rural/ urban residence* was determined using the census designation for each participant’s geographic region of residence and was defined using Ugandan Bureau of Statistics definitions [30]. *Loneliness* was measured with the 3-item UCLA Loneliness Scale, which asks participants about whether they “never,” “sometimes,” or “often” feel a lack of companionship, feel left out of community meetings or events, or feel isolated from others. The total score ranges from 3 to 9, with higher scores indicating a higher degree of loneliness [31]. As the scores were positively skewed in our sample, the top quintile was used to dichotomize responses, consistent with the literature [32], with a score of ≥ 5 being used to define loneliness. *Depressive symptoms* were measured using a version of the depression subscale of the Hopkins Symptom Checklist [33] modified for the Ugandan context [34, 35], with probable depression defined as a score > 1.75 [36]. *Anxiety symptoms* were measured using the General Anxiety Disorder (GAD)-7 scale [37]. We followed clinical categorizations of anxiety levels as follows: GAD-7 score of 0–4 (none), 5–9 (mild), 10–14 (moderate), and 15–21 (severe) and then further collapsed moderate and severe into a single category, due to the sparse data in each category. *Perceived stress* was measured using the Perceived Stress Scale [38], categorized into minimal stress (scores ranging from 0 to 13), moderate stress (scores 14–26), and high stress (scores 27–40).

### Potential Confounders and Other Variables

Income, sex, marital status, living alone, physical activity, alcohol consumption, and body mass index (BMI) were all considered as possible confounders. Income, sex assigned at birth, marital status, and living alone were measured based on self-report. BMI was computed using height and weight measurements, obtained by study staff. Physical activity was measured in metabolic equivalents (METs) per week, based on reported frequency of high, moderate, and low-intensity physical activity. Alcohol consumption was measured using the 3-item consumption subset of the Alcohol Use Disorders Identification Test (AUDIT-C) [39] and was classified as low vs. moderate-heavy alcohol use. For PWH, we also obtained information on ART regimen, viral load, and CD4 + T cell count for descriptive purposes.

### Statistical Analysis

The present analysis included only study participants with complete information on the three measures of sleep health (*n* = 556). We first compared the variables by HIV serostatus using Student’s *t*-tests, chi-squared tests, or analysis of variance as appropriate. To estimate the association between the explanatory variables and sleep outcomes, we fitted three separate multivariable models for each sleep outcome. Logistic regression was used for the binary outcomes of sleep quality and sleep efficiency and linear regression was used for sleep duration. Confounders found to be significantly (*p* < 0.05) related to each sleep outcome in bivariate analyses were adjusted for in the multivariable regression models. All regression models included explanatory variables of interest, guided by our conceptual framework. All analyses were conducted using SAS software, version 9.4 (SAS Institute Inc., Cary, NC, USA).

## Results

Characteristics of the study sample are shown in Table 1. Of the 556 study participants, almost half (*n* = 271) were PWH 50.09% were women (*n* = 279), most were married (*n* = 360), and 40.87% lived in an urban area (*n* = 225). Less than a tenth of the total sample were categorized as lonely (*n* = 51), a quarter had probable depression (*n* = 131), 27.85% had mild or moderate symptoms for anxiety (*n* = 154), and 73.20% reported moderate levels of perceived stress (*n* = 407).

**Table 1:** Demographic Characteristics of the Sample

	All	People with HIV (*n* = 271)	People without HIV (*n* = 285)	*P*-value
	N (%)			0.73
**Sex**				
Male	287 (49.91%)	133 (49.11%)	144 (50.68%)	
Female	288 (50.09%)	138 (50.89%)	141 (49.32%)	
**Dwelling**				0.12
Rural	340 (59.13%)	160 (58.01%)	171 (60.20%)	
Urban	235 (40.87%)	111 (41.99%)	114 (39.80%)	
**Marital Status**				**< 0.001**
Single	16 (2.79%)	8 (2.85%)	7 (2.73%)	
Married/ Cohabitating	372 (64.81%)	138 (50.60%)	222 (77.47%)	
Divorced/ Separated	49 (8.54%)	32 (11.74%)	16 (5.46%)	
Widowed	137 (23.87%)	93 (33.81%)	39 (14.33%)	
**Living Arrangement**				**0.003**
Live Alone	36 (6.26%)	25 (9.25%)	9 (3.40%)	
Live with Others	539 (93.74%)	246 (90.75%)	276 (96.60%)	
**Alcohol Use**				0.19
Low Risk		242 (89.3%)	244 (85.6%)	
Moderate-High Risk		29 (10.7%)	41 (14.4%)	
**Depression**				.**05**
Under clinical threshold	403 (75.47%)	188 (71.76%)	215 (79.04%)	
At or over clinical threshold	131 (24.53%)	74 (28.24%)	57 (20.96%)	
**Anxiety**				0.10
No symptoms	399 (72.15%)	192 (70.85%)	207 (73.40%)	
Mild symptoms	134 (24.23%)	73 (26.94%)	61 (21.63%)	
Moderate to severe symptoms	20 (3.62%)	6 (2.21%)	14 (4.96%)	
**Loneliness**				**0.003**
Below clinical threshold	505 (90.83%)	236 (87.08%)	269 (94.39%)	
At or above clinical threshold	51 (9.17%)	35 (12.92%)	16 (5.61%)	
**Percieved Stress**				0.12
Mild symptoms	142 (25.54%)	65 (23.99%)	77 (27.02%)	
Moderate symptoms	407 (73.20%)	205 (75.65%)	202 (70.88%)	
High symptoms	7 (1.26%)	1 (0.37%)	6 (2.11%)	
**HIV Viral Load**				-
Undetectable viral load		152 (57.14%)	-	
HIV-1 RNA < 40 cc/mL		65 (24.44%)	-	
HIV-1 RNA ≥ 40 cc/mL		49 (18.42%)	-	
**ART Regimen**				
3tc + azt + dtg		2 (0.75%)		
3tc + tdf + atv + rtv		7 (2.64%)		
3tc + tdf + dtg		243 (91.70%)		
3tc + tdf + efv		7 (2.64%)		
Other		6 (2.26%)		
**Sleep Efficiency**				0.19
* ≥*85%	352 (63.31%)	179 (66.05%)	173 (60.70%)	
<85%	204 (36.69%)	92 (33.95%)	112 (39.30%)	
**Sleep Quality**				0.35
Good	457 (82.19%)	227 (83.76%)	230 (80.70%)	
Poor	99 (17.81%)	44 (16.24%)	55 (19.30%)	
		**Mean** ***(SD)***		
**Age**	59.29 (6.45)	59.21 (6.06)	59.55 (6.80)	0.54
**BMI**	24.00 (5.07)	23.80 (4.68)	24.20 (5.41)	0.35
**Physical Activity in METs**	12,893.91 (7,451.24)	12040.39 (7192.20)	13711.23 (7614.42)	**0.01**
**Depressive Symptoms**	1.54 (0.41)	1.58 (0.43)	1.49 (0.38)	**0.01**
**Anxiety**	2.88 (3.13)	2.80 (2.91)	2.96 (3.32)	0.54
**Loneliness**	3.62 (1.09)	3.77 (1.23)	3.47 (0.92)	**0.001**
**Perceived Stress**	16.02 (4.43)	16.36 (3.55)	15.69 (5.11)	0.07
**Sleep Duration**	6.46 (1.74)	6.43 (1.78)	6.49 (1.71)	0.71
**CD4 + T Cell Count**	-	573.49 (203.93)	-	-
		**Median** ***(IQR)***		
Average Income*	100k (50k-1,000k)	150k (50k-1,000k)	100k (60k -300k)	0.26

Notes. BMI = Body mass index; METS = metabolic equivalents; *over past 3 months

PWH were more likely to be divorced/separated or widowed (45.55% vs. 19.79%, *p* < 0.001), live alone (9.25% vs. 3.40%, *p* = 0.0028), report be lonely (12.92%% vs. 5.61%%, *p* = 0.0029), and report lower physical activity (12,040.439 vs. 13,711.23 METs/week, *p* = 0.0082) compared to PWoH. Most PWH had undetectable viral loads (defined as < 40 copies/ml, *n* = 217, 81.58%) and the mean CD4 cell count was 573.49 (SD = 203.93). All PWH were currently taking ART and the majority (91.64%) were on Tenofovir, Lamivudine, and Dolutegravir (TLD).

### Sleep Health Outcomes by HIV Serostatus

Most individuals reported very good (*n* = 182; 33%) or fairly good (*n* = 274; 49%) sleep quality. The mean sleep duration was 6.46 h (*SD* = 1.78), with nearly half of the sample reporting 7–8 h of sleep (49.37%). The mean sleep efficiency was 73.98% (*SD* = 19.52%), with 36.69% (*n* = 204) reporting sleep efficiency at or above 85%. There were no statistically significant differences in sleep health by HIV serostatus: compared to PWoH, PWH had a similar distribution of sleep quality (83.76% vs. 80.70%; i.e., reporting good sleep, *p* = 0.303), sleep duration (mean 6.43 h vs. 6.48 h, *p* = 0.775), or sleep efficiency (33.95% vs. 39.30%, *p* = 0.338).

### Correlates of Sleep Health

In bivariate analyses, living in an urban location (*p* = 0.041), loneliness *(p* = 0.002), depressive symptoms (*p* < 0.0001), and anxiety symptoms (*p* < 0.0001) were associated with sleep quality; stress (*p* = 0.025), depressive symptoms (*p* < 0.0001), living alone (*p* < 0.0001), older age (*p* = 0.0046) were associated with sleep duration; depressive symptoms (*p* = 0.0047), BMI (*p =* 0.032), and physical activity (*p* = 0.031) were associated with sleep efficiency.

In a multivariable logistic regression, good sleep quality was inversely associated with having a positive screen for depression (adjusted odds ratio [aOR] = 0.21; 95% CI: 0.12, 0.36) (Table 2). In multivariable linear regression, sleep duration was inversely associated with having a positive screen for depression (b=-0.44; 95% CI:-0.60, 0.-0.28) and positively associated with age (b = 0.02, 95% CI: 0.009, 0.030) (Table 3). In multivariable logistic regression, optimal sleep efficiency was inversely associated with having a positive screen for depression (aOR = 0.51; 95% CI: 0.31, 0.83) and positively associated with BMI (aOR = 1.05; 95% CI: 1.01, 1.08) (Table 4).

**Table 2:** Logistic Regression on Good Sleep Quality

Variable	AOR	95% CI	*P*-value
Age	1.01	(0.97, 1.05)	0.52
HIV infection	1.40	(0.85, 2.29)	0.19
Urban Residence	1.53	(0.91, 2.56)	0.11
Probable Depression	0.21	(0.12, 0.36)	**< 0.0001**
Loneliness	1.08	(0.50, 2.35)	0.84
Moderate versus Minimal Stress	1.38	(0.79, 2.43)	0.50
High versus Minimal Stress	0.91	(0.11, 7.36)	0.81
Mild versus Minimal Anxiety Symptoms	0.49	(0.28, 0.84)	0.27
Moderate-Severe versus Minimal Anxiety Symptoms	0.52	(0.17, 1.61)	0.60

**Table 3:** Linear Regression on Sleep Duration

Variable	b	SE	*P*-value
Age	0.02	0.0052	**0.0002**
HIV infection	-0.00079	0.066	0.99
Urban Residence	0.0030	0.067	0.96
Loneliness	0.073	0.12	0.56
Probable Depression	-0.44	0.083	**< 0.001**
Moderate versus Minimal Stress	-0.048	0.077	0.54
High versus Minimal Stress	0.24	0.35	0.49
Mild versus Minimal Anxiety Symptoms	-0.061	0.080	0.45
Moderate-Severe versus Minimal Anxiety Symptoms	0.18	0.19	0.34
Living alone	0.062	0.14	0.65

Notes. Further adjusted for living alone, due to significance at bivariate level

**Table 4:** Logistic Regression on Optimal Sleep Efficiency

Variable	AOR	95% CI	*P*-value
Age	1.02	(0.99. 1.05)	0.15
HIV infection	0.93	(0.64, 1.35)	0.72
Urban Residence	1.18	(0.81, 1.71)	0.39
Loneliness	0.80	(0.38, 1.68)	0.56
Probable Depression	0.51	(0.31, 0.83)	**0.006**
Moderate versus Minimal Stress	0.73	(0.48, 1.12)	0.80
High versus Minimal Stress	0.39	(0.04, 3.91)	0.50
Mild versus Minimal Anxiety	1.12	(0.71, 1.75)	0.58
Moderate-Severe versus Minimal Anxiety	1.79	(0.62, 5.15)	0.32
BMI	1.05	(1.01, 1.08)	**0.02**
Physical Activity	1.00	(1.00, 1.00)	0.05

Notes. Further adjusted for BMI and physical activity due to significance at bivariate level

.

## Discussion

Sleep affects health and quality of life of older adult populations with and without HIV. However, little is known whether HIV serostatus affects sleep differently in older people in Uganda or the correlates of sleep health in this population. In this cross-sectional study of older-age PWH on stable ART and PWoH in rural Uganda, we found that PWH and PWoH had similar rates of sleep health outcomes. This finding contradicted our hypothesis and also contrasts to the existing literature from middle- and high-income countries demonstrating a higher burden of sleep problems among PWH compared to PWoH [3, 4, 40, 41]. We speculate that the PWH in our sample may be more resilient, with less vulnerability to poor sleep health, compared with PWH residing in middle- and high-income countries. All PWH in our sample were on ART (specifically on TLD), had viral suppression, and had a mean CD4 T cell count greater than 500, indicating that their HIV is well managed. Given the high levels of viral suppression, HIV-associated sleep disturbances may have been minimized. Over 90% of our sample with HIV were on a TLD-based regimen, suggesting that the patterns observed may be most generalizable to those on this regimen.

Most of our study participants reported very or fairly good sleep quality and had optimal sleep duration. However, only a third of our sample had optimal sleep efficiency. According to studies conducted in high-income countries, poor objective and subjective sleep efficiency, rather than the perceived and actual sleep duration, correlates more strongly with adverse health outcomes including cognitive impairment and cardiometabolic conditions [42, 43]. Sleep efficiency is one of the few sleep parameters that declines with age [44]. It is therefore imperative to assess the concordance between objective and subjective sleep efficiencies in this population to better understand the epidemiology and predictors of sleep efficiency.

Of the explanatory variables that we considered, depressive symptoms were the only variable that consistently associated with all sleep outcomes. This finding aligns with a large body of literature that shows significant associations between depressive symptoms and sleep health [45, 46]. No other variables were associated with all three measures of sleep health, suggesting that the management of depressive symptoms may be the most effective strategy in improving overall sleep health. It is important to note that our findings are not too surprising since sleep disturbance is part of the diagnostic nosology of depression. Nevertheless, interventions targeting depressive symptoms may improve sleep among older Ugandans with or without HIV. Longitudinal studies are warranted to determine the potential bidirectionality of the relationship and elucidate pathways that can explain the relationship between depressive symptoms and sleep health among older Ugandans. Further, consistent with previous reports [3, 41], we found that PWH exhibited higher levels of depressive symptoms and loneliness compared to PWoH. This highlights the crucial role of mental health in the etiology of sleep difficulties and also the importance of addressing mental well-being in older Ugandans with HIV.

Our study revealed other findings not consistent with existing work. For example, older age was associated with longer sleep duration in our sample, contrary to a large body of literature showing declining sleep duration with increasing age [47]. Our sample was relatively younger than the studies of older adults showing decreasing sleep duration, hence the potential reason for the conflicting findings. Of the confounders we considered, higher BMI was associated with improved sleep efficiency. However, the distribution of BMI in our sample was largely in the normal range. Thus, our finding may suggest that adequate nutrition is associated with improved sleep efficiency.

There are several limitations to consider when interpreting the findings. The use of a cross-sectional design in our study limits establishing causal relationships among the identified associations. Our reliance on self-reported sleep health outcomes, without the inclusion of objective measures derived from actigraphy or polysomnography, could have biased our estimates of the associations between sleep health and the explanatory variables of interest. However, the self-report measure of sleep health was sufficient to identify an association between probable depression. In addition, we lacked information about participants’ comorbidities including sleep disorders and different types of medications that can affect sleep health. Finally, the recruitment strategy centered around regional clinics may hinder the generalization of our findings to other older adults residing in different parts of Uganda. Despite these limitations, our large sample size, encompassing a significant number of older PWH and PWoH, allowed us to compare the differences between the groups. Additionally, the use of a theoretically grounded framework using the Sleep Senescent Model [16] provided an opportunity for deep phenotyping of sleep health compared to what has been reported previously, contexualized in this setting.

## Conclusions

This study sheds light on the prevalence and correlates of sleep outcomes in older Ugandans, both with and without HIV. Overall, we found a high proportion of respondents reporting good sleep quality and duration. Sleep outcomes did not significantly differ between PWH and PWoH. However, PWH exhibited higher levels of depressive symptoms and loneliness, highlighting the importance of addressing mental health in this population. Our findings underscore the important association between depression and various aspects of sleep health, emphasizing the need to embed mental health assessment into HIV clinical care to improve sleep and overall well-being of older Ugandans.
